# Seroprevalence of Rubella Virus-specific Antibodies in Women and the Diagnostic Efficacy of Enzyme-linked Immunoassay and Rapid Immunochromatographic Tests

**DOI:** 10.7759/cureus.7246

**Published:** 2020-03-12

**Authors:** Praveen R Shahapur, Venkataramana Kandi

**Affiliations:** 1 Microbiology, BLDE (Deemed to be University) Shri B.M. Patil Medical College, Vijaypur, IND; 2 Clinical Microbiology, Prathima Institute of Medical Sciences, Karimnagar, IND

**Keywords:** rubella virus, children, young adults, seroprevalence, diagnostic efficacy, enzyme linked immunosorbent assay (elisa), rapid immunochromatographic tests, pregnant women

## Abstract

Introduction

Rubella is an infectious disease caused by the Rubella virus. The disease was previously called German measles and is transmitted through respiratory aerosols. Rubella causes both clinical and subclinical infections in children and young adults. Rubella virus has teratogenic capabilities and may cause severe complications in the fetuses of women who acquire Rubella viral infection during their pregnancy. The present study aims to evaluate the seroprevalence of anti-Rubella virus immunoglobulin (Ig) G and IgM antibodies in both pregnant and non-pregnant women and assess the diagnostic efficacy of enzyme-linked immunosorbent assay (ELISA) and rapid immunochromatographic tests.

Methods

The study included 240 females in the age range of 16-45 years. The study subjects included both pregnant women and non-pregnant women. After informed consent, 5 milliliters of blood was collected from each participant, and serum was separated and tested for the presence of antibodies (IgG and IgM) against the Rubella virus using both the traditional ELISA (Delta Biologicals, Pvt. Ltd., China) and a rapid ELISA-immunochromatographic test (ICT) (Span Biotech. Ltd., China). The data collected were systematically entered into Microsoft Excel sheets (Microsoft Corporation, Redmond, Washington) and were analyzed using SPSS Statistics for Windows, Version 17.0, 2008 (SPSS Inc., Chicago, Illinois).

Results

The study revealed an overall seroprevalence of 31.66% for Rubella-specific IgG and IgM antibodies. Out of the 125 pregnant women included in the study, 49 (39.20%) were seropositive for Rubella IgG antibodies, and among the 115 non-pregnant women tested, 24 (20.86%) were positive for Rubella IgG antibodies. Four (5.26%) of the 76 seropositive women revealed IgM antibodies. The sensitivities of both the ELISA (40.61%) and rapid immunochromatographic (39.20%) tests were observed to be low and the specificities of both methods were similar (79.13%).

Conclusion

The seroprevalence of Rubella-specific IgG antibodies was observed to be low as compared to the other regions of India. The low seroprevalence may predispose pregnant women to Rubella viral infection and may lead to increased incidences of congenital Rubella syndrome (CRS). Both the ELISA and immunochromatographic tests showed low sensitivity and similar specificities.

## Introduction

Rubella is one of the many ribonucleic acid (RNA) viruses that infect humans [1]. Although Rubella belongs to the family of arboviruses (Togaviridae), it is transmitted to humans through the respiratory route. The infection caused by the Rubella virus is also called German measles owing to its discovery by German physicians. Rubella is a self-limiting viral infection frequently seen in children and is prevalent throughout the world [2]. Rubella viral infection results in fever and a maculopapular rash, which is similar in presentation to measles viral infection. Rubella virus can be transmitted from mother to child through the transplacental barrier when a mother acquires the infection in her first trimester of pregnancy.

Rubella virus has teratogenic capabilities, and it was observed that neonates infected with Rubella transplacentally suffer from congenital rubella syndrome (CRS). CRS may present as serious encephalitis, low-birth weight, thrombocytopenia, anemia, hepatitis, and premature delivery. It may also present as blindness, deafness, heart disease, and skin conditions [3]. There are several other reports that suggest that the women infected with Rubella virus during the early pregnancy may suffer from miscarriages, abortion, and stillbirth [4]. Before 1969, when the vaccine was not yet introduced, there were several reports of outbreaks of Rubella viral infection in children under 12 years of age [5]. After the inclusion of the Rubella vaccine in the immunization schedule, the incidences among the children have been very low. The cause of concern now is the infection in pregnant women. In most instances, these women could be either non-immunized or inadequately immunized.

The measles, mumps, and rubella(MMR) vaccine was available in India since 2000, but only recently, i.e., in 2017, the Rubella-containing vaccine was introduced in the National Immunization Schedule [6]. It, therefore, is imperative that the current Rubella vaccination is insufficient, and the virus may still be circulating among the population, which poses a threat to pregnant women and the fetus.

Traditionally, serological tests like Toxoplasma, Rubella, Cytomegalovirus, and the Herpes virus (TORCH) is used to screen for the presence of teratogenic viral infections in pregnant women with a history of spontaneous abortion, stillbirth, and congenital anomalies in new-born babies.

The present study is carried out to screen for the antibodies (IgG and IgM) against the Rubella virus in both pregnant and non-pregnant women using the traditional enzyme-linked immunosorbent assay (ELISA) and the rapid immunochromatography test (ICT).

## Materials and methods

The study included 240 female subjects with an age range of 16-45 years. Among the study participants, 125 were pregnant. Those who reached menopause were excluded from the study. An informed and written consent was obtained from all the study subjects and the study was approved by the institutional ethical committee of the BLDE Deemed University's Shri BM Patil Medical College (IEC Ref. No. 43/2013).

Five milliliters of blood was collected from each study participant. The serum was separated and used to test for the presence of antibodies (IgG and IgM) against the Rubella virus using the conventional ELISA (Delta Biologicals, Pvt. Ltd. China). All the samples were also tested for the presence of antibodies (IgG and IgM) against the Rubella virus using rapid ICT (Span Biotech. Ltd. China).

Statistical analysis

The data collected were systematically entered into the Microsoft Excel sheets (Microsoft Corporation, Redmond, Washington) and were analyzed using SPSS Statistics for Windows, Version 17.0, 2008, (SPSS Inc., Chicago, Illinois).

## Results

The Rubella seropositivity by the ELISA method was 31.66% as compared to the rapid assay, which revealed the total seropositivity of 30.41%. Out of the 125 pregnant women included in the study, 49 (39.20%) were seropositive for Rubella-specific IgG antibodies, and among the 115 non-pregnant women tested, 24 (20.86%) were positive for Rubella-specific IgG antibodies.

Four (5.26%) of the 76 seropositive women revealed IgM antibodies. Three (75%) out of the four IgM seropositive cases were detected by the ELISA method as compared to the rapid ICT method (25%). The comparative efficacy of the ELISA and rapid ICT test methods in screening for Rubella-specific IgG and IgM antibodies is shown in Table 1.

**Table 1:** Details of Rubella seropositivity and the diagnostic efficacy of the laboratory tests IgG: immunoglobulin G; IgM: immunoglobulin M; POS: positive; NEG: negative; PPV: positive predictive value; NPV: negative predictive value; ELISA: enzyme-linked immunosorbent assay; ICT: immunochromatographic test

Rubella Test	IgG POS	IgM POS	IgG & IgM NEG	Sensitivity	Specificity	PPV	NPV	ACCURACY
ELISA	73	3	164	40.61%	79.13%	45.48%	75.67%	67.58%
Rapid Test (ICT)	72	1	167	39.20%	79.13%	44.60%	75.23%	67.16%

## Discussion

Rubella is a contagious viral infection that is mostly seen in children under 15 years of age and young adults. Rubella is a vaccine-preventable viral infection that has no specific treatment. The infection is usually self-limiting but may result in serious complications in the new-born babies born to women infected with Rubella virus in their early pregnancy. Vaccination/immunization appears to be the only way to prevent infection, especially among pregnant women. Also, the vaccination among children will be instrumental in the elimination of the Rubella virus from the general population, thereby eliminating the probability of infection to the susceptible population, like pregnant women. 

The Rubella vaccine was not incorporated in the National Immunization Schedule (India) until recently. Although the MMR vaccine was available in India since 2010, it was not a part of the immunization program at birth [5]. Studies have also noted that the MMR immunization status among children was well below 50%. The Indian government, therefore, had included the Rubella vaccine in the National Immunization Program from 2017 [6].

The prevalence of Rubella-specific IgG antibodies was found to be 68.3% among non-vaccinated girls in the age group of 13-15 years as noted by a study from Kerala, South India. The same study had observed that the seropositive girls had unprotective Rubella-specific IgG antibody titers [7]. This makes them potential carriers of infection to the fetuses during pregnancy and the development of congenital Rubella syndrome (CRS).

A seroprevalence rate of 67.3% was noted among girls in the age group of 11-18 years as reported by a study from Jammu and Kashmir, North India [8]. The seroprevalence of Rubella-specific IgG antibodies appear to vary significantly with geographic locations, age, socioeconomic status, and the standards of living as evidenced by the reports from New Delhi, also a North Indian state with a 90% seroprevalence [9].

A seroprevalence of more than 90% both in pregnant and non-pregnant women was observed in a study from Nigeria [10]. A study from Turkey, which screened schoolgoing girls within the age range of 12-18 years and pregnant women in the age range of 26-35 years found a seroprevalence of 92.5%, and 100%, respectively, for Rubella-specific IgG antibodies by using a microparticle enzyme immunoassay [11]. This study, which was done among the non-vaccinated population, showed no relationship of socioeconomic status with Rubella seropositivity.

A post-vaccination survey among women in the age range of 12-42 years from Iran showed a 96% prevalence of Rubella-specific protective IgG antibodies [12]. A study from Zambia, which evaluated the seroprevalence of Rubella-specific IgG antibodies among female blood donors, noted seropositivity of 91.9% [13].

An evaluation of the presence of protective Rubella-specific IgG antibodies in pregnant women in Canada revealed that 87.6% had protective antibodies (>10 IU/mL), and 2.3% showed antibody titers <5 IU/mL (seronegative). This study found a positive relationship of seronegativity with educational qualifications where the university graduates had comparatively lower seronegativity rates (1.6%) as compared to other women in schools and colleges (3.1%) [14].

The World Health Organization (WHO) aims to improve the seropositivity rates of Rubella-specific IgG antibodies to 95% and limit the seronegativity rates to not >5% among pregnant women globally by the year 2020 [15].

A recent meta-analysis of the global prevalence of seronegativity of Rubella-specific IgG antibodies in women of child-bearing age (WCBA) that included five different WHO regions (Europe, Africa, America, Middle-East, and South-East Asia) revealed that all the studies from South-East Asia showed a seronegativity of >10% [16].

A study of the seroprevalence of Rubella-specific IgG antibodies among healthy pregnant women in China revealed that 83.3% of women had protective antibodies [17]. In a recent study from Cameroon, which evaluated the seroprevalence of Rubella-specific IgG antibodies among pregnant women found 94.4% seroprevalence. This study had hypothesized that the increased seroprevalence in the absence of adequate immunization could be attributed to the circulating wild strain of the Rubella virus [18].

A seroprevalence of 50% was recently reported from Kerala, South India. This study recruited 1671 non-vaccinated girls in the age range of 13-15 years and evaluated the Rubella-specific IgG antibody titers. The study revealed that among the seropositive girls, 50% had protective antibodies, 12% had an equivocal antibody titer, and 38% had low titers of antibodies [19].

In a meta-analysis of the seroprevalence of Rubella-specific IgG antibodies among women in Bangladesh showed a seropositivity range of 14 to 53% (seronegativity of 47% to 86%) [20]. A recent study from Pakistan, which included pregnant women, showed a seroprevalence of 16% and 2.5% to Rubella-specific IgG and IgM antibodies, respectively [21].

In a large-scale study from Nepal, which included more than 2000 women of childbearing age, a 90.8% seroprevalence was noted against Rubella-specific IgG antibodies [22]. In a population-based cross-sectional study reported from Tanzania, an economically constrained underdeveloped country, it was noted that the seroprevalence rates of anti-Rubella IgG antibodies were <5% [23].

In the current study, we have evaluated the seroprevalence of anti-Rubella IgG and IgM antibodies and assessed the diagnostic efficacy of the ELISA and rapid immunochromatographic tests in detecting Rubella-specific antibodies. The study included female subjects aged between 16 and 45 years and included both pregnant and non-pregnant women. The seropositivity rates for anti-Rubella IgM and IgG antibodies were noted to be 5.26% and 31.66%, respectively. The seropositivity among pregnant women (39.20%) was comparatively higher than among the non-pregnant women (20.86%).

The sensitivities of both ELISA (40.62%) and the rapid immunochromatographic methods (39.20%) were lower than the specificities, which were found to be the same for both the methods (79.13%). Low sensitivities of both the methods are an interesting observation, which indicates that there is an increased possibility of false negatives, and, therefore, the seroprevalence may be marginally higher than observed results/values.

The prevalence rates of anti-Rubella antibodies in the present study were significantly lower than those observed in other parts of India [3-5]. The prevalence rates of anti-Rubella antibodies in this study correlated with studies reported from Bangladesh, a socioeconomically similar country like India [16]. Only a few studies, which included one from Pakistan (16%) and another from Tanzania (<5%), revealed lower seroprevalence rates than those observed in the present study [17,19].

## Conclusions

Although a vaccine is available, and it is now included in the National Immunization Schedule (India), there have been several reports of infection in pregnant women resulting in Congenital Rubella Syndrome (CRS). The low seroprevalence rates of anti-Rubella IgG and IgM antibodies in the current study points to the fact that there is an increased need for effective vaccination against Rubella in this part of India. From the results of the present study, it can be hypothesized that both the Rubella vaccine strain and the wild Rubella virus strains are less frequently circulating in this part of the world. Widespread vaccination among children may improve the seropositivity rates and, in turn, will confer protective immunity against Rubella viral infection and its complications in the future.
